# Supplementary material for: Depression, anxiety and stress among high school students: A cross-sectional study in an urban municipality of Kathmandu, Nepal
Source: PLOS Glob Public Health. 2022 May 31;2(5):e0000516. doi: 10.1371/journal.pgph.0000516 (PMC10022099; doi:10.1371/journal.pgph.0000516)
Supplement: S1 File — (PDF) [file pgph.0000516.s001.pdf]

निर्देशन: कृपया उपयुक्त प्रतिक्रियामा गोलो लगाउनुहोस् र खाली ठाँऊ भएमा आफनो उत्तर खुलाउनुहोस् । कैफियत लेखेका जानकारीहरुलाई राम्ररी पढेर उत्तर दिनुहोस् ।-

Instruction: Please circle the appropriate response and fill up in blank spaces.

Note: Read carefully before answering.

भाग (क) Section A

सामाजिक तथा जनसाङ्ख्यिक, पारिवारिक र पढाईबारे जानकारी

Socio- demographic, familial and academic characteristics

क्र. स.	प्रश्नहरु (Questions)	प्रतिकयाहरु (Responses)	
१	लिङ्ग (Sex)	पुरुष (Male)<br>महिला (Female)<br>अन्य (others)	१<br>२<br>०
२	उमेर (Age)	…………….. वर्ष<br>कैषियत पूरा भएको वर्ष लेख्नुहोला	
३	विद्यालयको प्रकार (Type of school)	सरकारी /सामुदायिक (Public)<br>निजी / संस्थागत (Private)	१<br>२
४	तपाई हाल कुन संकायमा पढ्नुभइरहेको छ ? (Which stream do you study)	विज्ञान (Science)<br>व्यवस्थापन (Management)<br>मानविकी (Humanities)<br>अन्य (others)<br>यदि अन्य भए, खुलाउनुहोस् (If others then specify)<br>………………………………	१<br>२<br>३<br>४
५	तपाई कुन कक्षामा पढ्नुहुन्छ ? In which class do you study?	११ (11)<br>१२ (12)	१<br>२

६	पछिल्लो परीक्षामा आएको नतिजा<br>(Result in last examination)	अनुत्तिर्ण (Failed)<br>उत्तिर्ण (Passed)<br>यदि उत्तिर्ण भए (if passed then) ……………… (प्रतिशत / ग्रेण खुलाइदिनुहोस् (% or grade)	१<br>२
७	परिवारको किसिम ?<br>Family type	एकल - Nuclear (parents, son /daughter)<br>संयुक्त - Joint (parents, grandparents, son/daughter<br>बृहत - Extended (parents, grandparents,son/daughter,uncles/aunts/cousins)	१<br>२<br>३
८	बुबाको शैक्षिक योग्यता<br>Father's education	निरक्षर (Illiterate)<br>नेपाली भाषामा लेख्न पढ्न मात्र आउने (can only read and write in Nepali)<br>प्राथमिक तह (कक्षा ५ सम्म) (Primary)<br>माध्यमिक तह (कक्षा ६ - १०) (Secondary)<br>प्रविणता तह (कक्षा ११-१२) (Higher secondary)<br>स्नातक तह वा माथि (Bachelor's and above)	१<br>२<br>३<br>४<br>५<br>६
९	आमाको शैक्षिक योग्यता<br>Mother's education	निरक्षर (Illiterate)<br>नेपाली भाषामा लेख्न पढ्न मात्र आउने (can only read and write in Nepali)<br>प्राथमिक तह (कक्षा ५ सम्म) (Primary)<br>माध्यमिक तह (कक्षा ६ - १०) (Secondary)<br>प्रविणता तह (कक्षा ११-१२) (Higher secondary)<br>स्नातक तह वा माथि (Bachelor's and above)	१<br>२<br>३<br>४<br>५<br>६
१०	तपाई हाल कोसँग बस्नुहुन्छ ?<br>Who are you staying with currently?	परिवारसँग Staying with parents<br>नातेदारसँग बसेको Staying with relatives<br>छात्रावासमा बसेको Staying in hostel<br>साथीहरुसँग बसेको Staying with friends<br>श्रीमान् श्रीमतीसँग बसेको Staying with husband/wife<br>अन्य Others	१<br>२<br>३<br>४<br>५<br>६

		**यदी अन्य भए खुलाउनुहोस्** If others then please specify	

भाग (ख) Section B

सामाजिक घुलमिल (Socializing)

१२	तपाईसँग कति नजिकका साथी छन् ?<br>How many close friends do you have?	५ अथवा सोभन्दा बढि (Five or more friends)<br>५ भन्दा कम (Fewer than five friends.)	१<br>२
१३	तपाई दिनमा कति समय आफ्ना साथीहरुसँग घुलमिल गर्नुहुन्छ ?<br>What is the amount of time you spent socializing per day?	२ घण्टा अथवा बढि प्रतिदिन (Two or more hours per day.)<br>२ घण्टा भन्दा कम (Less than two hours per day.)	१<br>२

बुलिङ् (Bullying)

बुलिङ् भनेको जब एक वा बढि विद्यार्थीले अर्को विद्यार्थीलाई जिस्काउने, धम्काउने, अफवाह फैलाउने, हिर्काउने, धकेल्ने र बारम्बार दुख दिने गर्छन् ।
जब दुई उतिकै शक्तिशाली र बलियो विद्यार्थीहरु मिलनसार तरिकाले एक अर्कासँग बहस वा झगडा वा जिस्किने गर्छन् , त्यसलाई बुलिङ भनिन्न ।

Note: Bullying is when one or more student's tease, threaten, spread rumors about, hit, shove or hurt another student over and over again. It is not bullying when two students of about same strength or power argue or fight or tease each other in a friendly way.

१५	विगतको १२ महिनामा के तपाईलाई कसैले फोनमा सन्देश पठाएर अथवा इन्स्टाग्राम, फेसबुक वा अन्य सामाजिक संजाल मार्फत बुलिङ गरेको छ ?	छ (Yes)<br>छैन (No)	१<br>२

	During the past 12 months, have you ever been electronically bullied? (Count being bullied through texting, Instagram, facebook, or other social media)		

पढाईसँग सम्बन्धित तनाव बारे **(Academic Stress)**

१६	१ देखि ५ सम्म , तपाई तलको विवरणदेखि कतिको सहमत हुनुहुन्छ ?<br>**म मेरो पढाइको कारण निकै चिन्तित भएको अनुभव गर्छु ।**<br>On a scale of 1-5 how much do you agree with the following statement.<br>**I feel very stressed due to my studies.**	पूर्ण सहमत (Strongly agree)<br>सहमत (Agree)<br>न सहमत न असहमत (Neutral)<br>असहमत (Disagree)<br>पूर्ण असहमत (Strongly disagree)	१<br>२<br>३<br>४<br>५

भाग ग) Section- C

उदासिनता, चिन्ता, तनाव मापन (Depression, anxiety and stress scale)

कृपया हेरक विवरण ध्यानपूर्वक पढ्नुहोस् अनि ०,१,२ अथवा ३ मा गोलो धर्का खिच्नुहोस जसले विगतको **एक हप्तामा** यी विवरण तपाईमाथि कतिको लागु भयो भन्ने संकेत दिन्छ । कुनै प्रश्नको सहि वा गलत उत्तर छैन। यो तपाईको पछिलो अनुभव जान्नको लागि मात्र हो । कुनै पनि विवरणमा ज्यादा समय नफाल्नुहोस् ।

Please read each statement and circle a number 0, 1, 2 or 3 which indicates how much the statement applied to you over the past week. There are no right or wrong

answers. Do not spend too much time on any statement, but please answer each question.The rating scale is as follows:

० = मलाई पटक्कै लागू भएन (Did not apply to me)
१ = कुनै कुनै समय मलाई लागू भयो (Applied to me to some degree, or some of the time)
२ = धेरै समयसम्म मलाई लागू भयो (Applied to me to a considerable degree or a good part of time)
३ = पूर्ण रुपमा वा प्राय जस्तो समय मलाई लागू भयो (Applied to me very much or most of the time)

१	मलाई तनावमुक्त हुन गहारो लाग्यो । I found it hard to wind down	०	१	२	३
२	मलाई मेरो मुखको सूखापनबारे थाहा थियो । I was aware of dryness of my mouth	०	१	२	३
३	मैले कुनै पनि सकारात्मक भावना अनुभव गर्न सकिन । I couldn't seem to experience any positive feeling at all	०	१	२	३
४	मैले सास फेर्न अप्ठ्यारो भएको महसूस गरे - (जस्तै सामान्यभन्दा तीव्र गतिले सास फेर्नु, शारिरिक परिश्रम बिना पनि सास रोकिनु) I experienced breathing difficulty (e.g.) excessive rapid breathing, breathlessness in absence of physical exertion.	०	१	२	३
५	मैले कुनै पनि नयाँ कुरो आफै शुरु गर्न गहारो परेको अनुभव गरे । I found it difficult to work up the initiative to do things.	०	१	२	३
६	मैले कतिपय परिस्थितिहरुमा चाहिनेभन्दा बढि प्रतिकिया व्यक्त गरे । I tended to over-react to situations.	०	१	२	३

७	मैले आफू काँपेको महसूस गरें (जस्तै हातमा) ।<br>I experienced trembling (e.g. in the hands)	०	१	२	३
८	म धेरै अत्तालिएको मलाई अनुभव भयो ।<br>I felt that I was using of nervous energy	०	१	२	३
९	म डराउने अनि आफैलाई मुर्ख ठान्ने स्थितिहरुको बारे चिन्तित थिए ।<br>I was worried about situations in which I might panic and make a fool of myself	०	१	२	३
१०	मैले आशावादि हुनुपर्ने केहि कारण देखिन ।<br>I felt that I had nothing to look forward to	०	१	२	३
११	मैले आफूलाई अशान्त भएको पाँए ।<br>I found myself getting agitated	०	१	२	३
१२	मलाई आराम गर्न अप्ठ्यारो भएको महसूस भयो ।<br>I found it difficult to relax	०	१	२	३
१३	म अतिनै दुःखी भएको महसूस गरे ।<br>I felt down hearted and blue	०	१	२	३
१४	मैले गर्दै गरेको कुरामा बाधा पर्दा मेरो लागि असहनीय भयो ।	०	१	२	३

	I was intolerant of anything that kept me from getting on with what I was doing				
१५	म डराउन लागेको थिए भनी मैले थाहा पाएँ ।<br>I felt I was close to panic	०	१	२	३
१६	म कुनै पनि कुरोको विषयमा उत्साहित हुन सकिन ।<br>I was unable to become enthusiastic about anything	०	१	२	३
१७	म एक अति अयोग्य व्यक्ति रहेछु जस्तो मलाई लाग्यो ।<br>I felt I wasn't worth much as a person	०	१	२	३
१८	म अतिनै कमजोर जस्तो मलाई लाग्यो ।<br>I felt that I was rather touchy	०	१	२	३
१९	शारिरिक परिश्रमको अभावमा पनि आफनो हृदयको धड्कन महसूस गरे (तीव्र गतिले मुटु धडकिनु वा कहिले रोकिनु) ।<br>I was aware of the action of my heart in the absence of physical exertion (e.g. sense of heart rate increase, heart missing a beat)	०	१	२	३
२०	मलाई कुनै कारणबिनानै डर लागेको महसूस गरे ।<br>I felt scared without any good reason	०	१	२	३
२१	मैले आफनो जीवन अर्थहीन भएको महसूस गरे ।<br>I felt that life was meaningless	०	१	२	३

Thank you for your participation

सहभागिताको लागि धन्यवाद '
