# Supplementary material for: Depression, anxiety and stress among high school students: A cross-sectional study in an urban municipality of Kathmandu, Nepal
Source: PLOS Glob Public Health. 2022 May 31;2(5):e0000516. doi: 10.1371/journal.pgph.0000516 (PMC10022099; doi:10.1371/journal.pgph.0000516)
Supplement: Attachment — Submitted filename: response to reviewers .docx [file pgph.0000516.s002.docx]

Reviewer #1: This study looking at the prevalence and risk factors for common mental distress among adolescents has its significance as it tries to address a field that has limited studies in this geographical area. I think this manuscript and study could be strengthened.
There are some major concerns that I would like to share with the authors for improvements:

1. The author has written an introduction clearly providing the aim of the study. However, I would like to suggest the author look into recent studies highlighting the current prevalence of proposed outcome variables as many studies have been published since 2015-16. Furthermore, the overall prevalence of mental disorders among Nepalese adolescents provided by the National Mental Health Survey, Nepal-2020 has been reported in the discussion session, but I think it would be better to have it as an introduction as it covers a broader perspective.

- As per the suggestions received, the recent references have been added in the introduction portion. Also, the findings of National Mental Health Survey Nepal-2020 have been updated.

2. In methodology, a study from India has been used as a reference for sample size estimation. I wonder why the author didn’t look for any Nepalese past prevalence. Moreover, the reference study has provided the proportion for depression, anxiety, and stress while only the proportion of anxiety has been used by the author for sample estimation. Was the proportion of anxiety selected purposively to obtain optimal sample size or was there any other reason to ignore the provided prevalence of depression and stress? I suggest the author can aim to make the sample estimation section more explanatory and clearer.

- Prevalence was taken from a similar study conducted in India which had used the similar methodology and used DASS-21 to assess depression, anxiety and stress among the high school students. The reference study was conducted in Manipur, which has a similar setting to ours. Comparatively, the studies conducted in Nepal that had been published prior to conduct of our study either only assessed depression or was conducted at a single higher secondary school only and used different tool. Further, none of the studies had studied stress symptoms. Therefore, the most optimal reference study for estimation of sample size was taken to be study conducted in Manipur, India with similar socio-cultural setting as the study site of our study. The prevalence of anxiety was purposely selected to obtain the optimal sample size as the its proportion was larger compared to depression and stress which would give the largest sample size.
  - (Source: Kumar KS, Akoijam BS. Depression, Anxiety and Stress Among Higher Secondary School Students of Imphal, Manipur. Indian J Community Med. 2017;42: 94. doi:10.4103/IJCM.IJCM_266_15)

3. Is there any specific reason that the authors set the level of significance at 15% for bivariate analysis while the AOR has been interpreted at a 5% level of significance?

- The level of significance was set at 0.05 for both univariate and multivariable logistic regression. However, we selected the variables which had p value less than 0.15 in univariate analysis to be included in the multivariable logistic regression. It was done so that the important variables which were not significant at traditional level of significance (0.05) could also be included in the model.
- The goodness of fit of this regression model was tested by the application of the Hosmer and Lemeshow test; the model was found to be a good fit (P >.05). So, the authors decided to proceed with this model.
- Moreover, a study conducted by Bursac et al., recommended that in order to improve the chances of retaining meaningful confounders, the confounding level to 15% and the non-candidate inclusion level to 0.15 can be set.

Source: Bursac, Z., Gauss, C.H., Williams, D.K., Hosmer, D.W., 2008. Purposeful selection of variables in logistic regression. Source Code Biol. Med. 2008 31 3, 1–8. https://doi.org/10.1186/1751-0473-3-17

4. Humanities and science are very diverse disciplines. Is there any specific reason that the author has grouped science and humanities while management as its counterpart? Moreover, other disciplines are mentioned in Table 2 which are merged during cross-tabulation without clear explanation. Furthermore, in the discussion author has tried to emphasize the complexity of science as a discipline but this interpretation seems misleading as humanities have also been accommodated in the same group in the current manuscript.

**🡪** The faculties of humanities, education and special law had less number of observations. Therefore, these faculties were merged into science/humanities faculty in order to obtain more precise estimates. Moreover, few lines have been added in the discussion part of the manuscript explaining the possible reasons for presence of depression symptoms among humanities students.

5. In the discussion section, it is nice that the author has mentioned the national prevalence of mental disorders among adolescents based on the National Mental Health Survey 2019-2020. However, the study has been referenced as a study from 2018 but the factsheet of this national survey was published in 2020 so the author might need to revise the reference accordingly.

🡪 The reference has been revised accordingly.

6. I recommended that the author revisit their references focusing on studies based on adolescents and/or high school students. Medical/health science students might have more diverse characteristics than high school students. The author could shape the discussion by revisiting recent studies which have focused on depression among higher secondary school adolescents in Nepal and the Asian region.

🡪 As per the suggestions, we have tried to include more recent studies that focused on depression, anxiety and stress among high school students in Nepal.

7. In reference, some of the references are not attainable/assessable for instance current reference 29. Cross verification of the reference is required

🡪 Cross verification of the references has been done.

Reviewer #2: This manuscript presents results from a cross-sectional survey of HS students in Kathmandu. Given the dearth of local epidemiological data, especially among young populations, this is an important study.

1. Given the variation in how “high school” is defined across the world, it will be helpful to describe it. It appears that 11th and 12th grades are included.

- In Nepal, high school students comprise of grade 11 and grade 12 students. In Nepal, the high school differs from lower schooling level since the students have the opportunity to enroll in specialized areas such as science, management, humanities and education. High school are also popularly known as 10+2. The definition has been updated in methodology section.

2. Please include that the validation paper [Ref 31] conducted construct validity against a life satisfaction scale. As such, the scale wasn’t validated against a systematic

- The above mentioned comments have been updated accordingly.

3. Perhaps relatedly, the overall prevalence is very high. Globally, community samples show 5% prevalence of depression but this study found >50% rate. While such rate may be possible, it is unusually high. Indeed, many community-based studies find a very wide range of prevalence. Additional context will be helpful to interpret these high rates. How close were upcoming examinations? Any other stressors that may have increased overall mood and anxiety? How common is it for HS students to be asked about these issues when they are in the classroom?

- While the prevalence of symptoms of DAS reported by our study corroborates with the existing literatures in Nepal, it is exceptionally high. One possible explanation for this could be that the data was collected at the beginning of academic session. The students in the eleventh grade were undergoing sudden transition from school life to high school life with regards to new friends, teachers, school environment, and daily schedules whereas the students in 12th grade were awaiting results of previous board exam. This anticipation and the added pressure faced by 12th grade students for tremendous pressure for tertiary education may have contributed to the high prevalence of symptoms of DAS among 12th grade students whereas the higher prevalence of symptoms of DAS among 11th grade students could be possibly explained by the inability to cope with the adjustment of sudden transition from school level to high school level.

4. Overall, the language needs to be edited from “depression” and “anxiety” to “symptoms of depression” or “depressive symptoms” to clarify that the survey instrument is not diagnostic and is picking up presence of symptoms.

- These suggestions have been taken into consideration and changes have been made accordingly in the manuscript.

**Note to editor:**

The references have been changed in the revised manuscript. Kindly go through the reference.

# References

1.	Patel V, Flisher AJ, Hetrick S, McGorry P. Mental health of young people: a global public-health challenge. Lancet. 2007;369: 1302–1313. doi:10.1016/S0140-6736(07)60368-7

2.	Adolescent mental health. [cited 27 Jul 2021]. Available: https://www.who.int/news-room/fact-sheets/detail/adolescent-mental-health

3.	World Health Organization. Regional Office for South-East Asia. Mental health status of adolescents in South-East Asia: evidence for action. New Delhi: World Health Organization. Regional Office for South-East Asia; 2017. Available: https://apps.who.int/iris/handle/10665/254982%0A

4.	World Health Organization (WHO). Investing in mental health. Investing in mental health. 2003. Available: https://apps.who.int/iris/handle/10665/42823

5.	Bhattarai D, Shrestha N, Paudel S. Prevalence and factors associated with depression among higher secondary school adolescents of Pokhara Metropolitan, Nepal: a cross-sectional study. BMJ Open. 2020;10: e044042. doi:10.1136/bmjopen-2020-044042

6.	Gautam P, Dahal M, Ghimire H, Chapagain S, Baral K, Acharya R, et al. Depression among Adolescents of Rural Nepal: A Community-Based Study. Depress Res Treat. 2021;2021. doi:10.1155/2021/7495141

7.	M. Bhandari. Anxiety and depression among adolescent students at higher secondary school. BIBECHANA. 2016;14: 113–129. doi:http://dx.doi.org/10.3126/bibechana.v14i0.16019

8.	Bhandari K, Adhikari C. Prevalence and Factors Associated with Anxiety Disorder among Secondary School Adolescents of Dang District , Nepal. In: Journal of Gandaki Medical College - Nepal [Internet]. 2015 [cited 2 Jan 2022] pp. 53–56. Available: www.gmc.edu.np

9.	Sharma P, Choulagai B. Stress, anxiety, and depression among adolescent students of public schools in Kathmandu. [cited 19 Mar 2022]. Available: www.jiom.com.np

10.	Nepal Health Research Council. National Mental Health Survey, Nepal-2020. In: Nepal Health Research Council [Internet]. 2020 pp. 1–4. Available: http://nhrc.gov.np/projects/nepal-mental-health-survey-2017-2018/

11.	Kumar Aryal K, Bista B, Bahadur Khadka B, Raj Pandey A, Mehta R, Kumar Jha B, et al. Global School Based Student Health Survey Nepal-2015. Nepal Health Research Council; 2017. Available: http://nhrc.gov.np/wp-content/uploads/2017/10/Ghsh-final-with-cover-and-anex.pdf

12.	Kumar KS, Akoijam BS. Depression, Anxiety and Stress Among Higher Secondary School Students of Imphal, Manipur. Indian J Community Med. 2017;42: 94. doi:10.4103/IJCM.IJCM_266_15

13.	Jha KK, Singh SK, Nirala SK, Kumar C, Kumar P, Aggrawal N. Prevalence of Depression among School-going Adolescents in an Urban Area of Bihar, India. Indian J Psychol Med. 2017;39: 287–292. doi:10.4103/0253-7176.207326

14.	Bhasin SK, Sharma R, Saini NK. Depression, anxiety and stress among adolescent students belonging to affluent families: a school-based study. Indian J Pediatr. 2010;77: 161–165. doi:10.1007/s12098-009-0260-5

15.	Buabbas AJ, Hasan H, Buabbas MA. The associations between smart device use and psychological distress among secondary and high school students in Kuwait. PLoS One. 2021;16: e0251479. doi:https://doi.org/10.1371/journal.pone.0251479

16.	Anjum A, Hossain S, Sikder T, Uddin ME, Rahim DA. Investigating the prevalence of and factors associated with depressive symptoms among urban and semi-urban school adolescents in Bangladesh: a pilot study. Int Health. 2019;00: 1–9. doi:10.1093/INTHEALTH/IHZ092

17.	Sandal RK, Goel NK, Sharma MK, Bakshi RK, Singh N, Kumar D. Prevalence of Depression, Anxiety and Stress among school going adolescent in Chandigarh. J Fam Med Prim Care. 2017;6: 405. doi:10.4103/2249-4863.219988

18.	Ang AL, Wahab S, Rahman FNA, Hazmi H, Yusoff RM. Depressive symptoms in adolescents in Kuching, Malaysia: Prevalence and associated factors. Pediatr Int. 2019;61: 404–410. doi:10.1111/PED.13778

19.	Rodrigo C, Welgama S, Gurusinghe J, Wijeratne T, Jayananda G, Rajapakse S. Symptoms of anxiety and depression in adolescent students; a perspective from Sri Lanka. Child Adolesc Psychiatry Ment Heal 2010 41. 2010;4: 1–3. doi:10.1186/1753-2000-4-10

20.	Perren S, Dooley J, Shaw T, Cross D. Bullying in school and cyberspace: Associations with depressive symptoms in Swiss and Australian adolescents. Child Adolesc Psychiatry Ment Health. 2010;4: 28. doi:10.1186/1753-2000-4-28

21.	Abbo C, Kinyanda E, Kizza RB, Levin J, Ndyanabangi S, Stein DJ. Prevalence, comorbidity and predictors of anxiety disorders in children and adolescents in rural north-eastern Uganda. Child Adolesc Psychiatry Ment Health. 2013;7: 21. doi:10.1186/1753-2000-7-21

22.	Nguyen DT, Dedding C, Pham TT, Wright P, Bunders J. Depression, anxiety, and suicidal ideation among Vietnamese secondary school students and proposed solutions: a cross-sectional study. BMC Public Health. 2013;13: 1195. doi:10.1186/1471-2458-13-1195

23.	Gurung M, Chansatitporn N, Chamroonsawasdi K, Lapvongwatana P. Academic Stress among High School Students in a Rural Area of Nepal: A Descriptive Cross-sectional Study. JNMA J Nepal Med Assoc. 2020;58: 306. doi:10.31729/JNMA.4978

24.	Fergusson DM, Woodward LJ. Mental Health, Educational, and Social Role Outcomes of Adolescents With Depression. Arch Gen Psychiatry. 2002;59: 225–231. doi:10.1001/ARCHPSYC.59.3.225

25.	Brief Introduction | Tokha Municipality, Office Of the Municipal Executive. [cited 25 Jul 2021]. Available: https://www.tokhamun.gov.np/en/node/4

26.	Tokha Municipality Profile | Facts & Statistics – Nepal Archives. [cited 25 Jul 2021]. Available: https://www.nepalarchives.com/content/tokha-municipality-kathmandu-profile/

27.	Nepal - Secondary Education - Level, Schools, Technical, and Training - StateUniversity.com. [cited 22 Mar 2022]. Available: https://education.stateuniversity.com/pages/1058/Nepal-SECONDARY-EDUCATION.html

28.	Charan J, Biswas T. How to Calculate Sample Size for Different Study Designs in Medical Research? Indian J Psychol Med. 2013;35: 121. doi:10.4103/0253-7176.116232

29.	Vankim NA, Nelson TF. Vigorous physical activity, mental health, perceived stress, and socializing among college students. Am J Health Promot. 2013;28: 7–15. doi:10.4278/ajhp.111101-QUAN-395

30.	2019 State and Local Youth Risk Behavior Survey. 2019; 7. Available: https://www.cdc.gov/healthyyouth/data/yrbs/pdf/2019/2019_YRBS-Standard-HS-Questionnaire.pdf

31.	Coker AO, Coker OO, Sanni D. Psychometric properties of the 21-item Depression Anxiety Stress Scale (DASS-21). African Res Rev. 2018;12: 135. doi:10.4314/afrrev.v12i2.13

32.	Tonsing KN. Psychometric properties and validation of Nepali version of the Depression Anxiety Stress Scales (DASS-21). Asian J Psychiatr. 2014;8: 63–66. doi:10.1016/j.ajp.2013.11.001

33.	Billah SMB, Khan FI. Depression among Urban Adolescent Students of Some Selected Schools. Faridpur Med Coll J. 2015;9: 73–75. doi:10.3329/FMCJ.V9I2.25678

34.	Zhang SC, Yang R, Li DL, Wan YH, Tao FB, Fang J. Association of health literacy and sleep problems with mental health of Chinese students in combined junior and senior high school. PLoS One. 2019;14: e0217685. doi:10.1371/journal.pone.0217685

35.	World Health Organization (WHO). Nepal WHO Special Initiative for Mental Health Situational Assessment CONTEXT.

36.	Alharbi R, Alsuhaibani K, Almarshad A, Alyahya A. Depression and anxiety among high school student at Qassim Region. J Fam Med Prim care. 2019;8: 504–510. doi:10.4103/jfmpc.jfmpc_383_18

37.	Mishra SK, Mona S, K TN. Prevalence of depression and anxiety among children in rural and suburban areas of Eastern Uttar Pradesh: A cross‑sectional study Shailendra. J Fam Med Prim Care. 2018;7: 21–26. doi:10.4103/jfmpc.jfmpc

38.	Mohammadi MR, Pourdehghan P, Mostafavi S-A, Hooshyari Z, Ahmadi N, Khaleghi A. Generalized anxiety disorder: Prevalence, predictors, and comorbidity in children and adolescents. J Anxiety Disord. 2020;73: 102234. doi:10.1016/j.janxdis.2020.102234

39.	Simegn WI, Dagnew BI, Yeshaw YI, Yitayih S, Woldegerima B, Dagne HI. Depression, anxiety, stress and their associated factors among Ethiopian University students during an early stage of COVID-19 pandemic: An online-based cross-sectional survey. 2021. doi:10.1371/journal.pone.0251670

40.	Reardon LE, Leen-Feldner EW, Hayward C. A critical review of the empirical literature on the relation between anxiety and puberty. Clin Psychol Rev. 2009;29: 1–23. doi:10.1016/j.cpr.2008.09.005

41.	Christiansen DM. Examining Sex and Gender Differences in Anxiety Disorders. A Fresh Look Anxiety Disord. 2015 [cited 9 Oct 2021]. doi:10.5772/60662

42.	Sahar N, Muzaffar NM. Role of Family System, Positive Emotions and Resilience in Social Adjustment among Pakistani Adolescents. J Educ Heal Community Psychol. 2017;6: 46. doi:10.12928/jehcp.v6i2.6944

43.	Arif N, Yadav S, Gupta S. An epidemiological study of stress among adolescent of urban and rural Moradabad. Int J Med Sci Public Heal. 2019;8: 1. doi:10.5455/ijmsph.2019.0512915062019

44.	Luthar SS. The culture of affluence: psychological costs of material wealth. Child Dev. 2003;74: 1581–1593. doi:10.1046/j.1467-8624.2003.00625.x

45.	Luthar SS, D’Avanzo K. Contextual factors in substance use: a study of suburban and inner-city adolescents. Dev Psychopathol. 1999;11: 845–867. doi:10.1017/s0954579499002357

46.	Yusoff MSB, Abdul Rahim AF, Baba AA, Ismail SB, Mat Pa MN, Esa AR. Prevalence and associated factors of stress, anxiety and depression among prospective medical students. Asian J Psychiatr. 2013;6: 128–133. doi:10.1016/j.ajp.2012.09.012

47.	Mikulincer M, Shaver PR, Pereg D. Attachment Theory and Affect Regulation: The Dynamics, Development, and Cognitive Consequences of Attachment-Related Strategies. Motiv Emot. 2003;27: 77–102. doi:10.1023/A:1024515519160

48.	Chokshi AS, Rangwala PP, Dumra GH, Thakrar MR, Singh AJ, Lakdawala BM. Depression, anxiety and stress amongst students in science verses non-science stream: a comparative study. Int J Community Med Public Heal. 2021;8: 3461. doi:10.18203/2394-6040.IJCMPH20212602

49.	Baviskar M, Edward K, Hospital M, Instit P. Depression , Anxiety and stress : A comparative study in Arts , Science and Commerce students from a Rural area of India. 2013; 1–4.

50.	Waghachavare VB, Chavan VM, Dhumale GB, Gore a D. A Cross-Sectional Study of Stress Among Junior College Students in a Rural Area of Sangli District of Maharashtra . Innov J Med Heal Sci. 2013;3: 294–297.

51.	Pascoe MC, Hetrick SE, Parker AG. The impact of stress on students in secondary school and higher education. Int J Adolesc Youth. 2019; 1–9. doi:10.1080/02673843.2019.1596823

52.	Nixon CL. Current perspectives: the impact of cyberbullying on adolescent health. Adolesc Health Med Ther. 2014;5: 143. doi:10.2147/AHMT.S36456

53.	Bottino SMB, Bottino CMC, Regina CG, Correia AVL, Ribeiro WS. Cyberbullying and adolescent mental health: systematic review. Cad Saude Publica. 2015;31: 463–475. doi:10.1590/0102-311X00036114
